# Does quantitative lung SPECT detect lung abnormalities earlier than lung function tests? Results of a pilot study

**DOI:** 10.1186/s13550-014-0039-1

**Published:** 2014-08-01

**Authors:** Pernilla Norberg, Hans Lennart Persson, Birgitta Schmekel, Gudrun Alm Carlsson, Karl Wahlin, Michael Sandborg, Agnetha Gustafsson

**Affiliations:** Medical Radiation Physics, County Council of Östergötland, Linköping, 581 85 Sweden; Center for Medical Image Science and Visualization (CMIV), Linköping University, Linköping, 581 83 Sweden; Department of Respiratory Medicine and Department of Medical and Health Sciences, Linköping University, Linköping, 581 83 Sweden; Department of Clinical Physiology and Department of Medical and Health Sciences, Linköping University, Linköping, 581 83 Sweden; Department of Medical and Health Sciences, Linköping University, Linköping, 581 83 Sweden; Department of Computer and Information Science, Linköping University, Linköping, 581 83 Sweden; Department of Medical Physics, Karolinska University Hospital, Huddinge, Stockholm 141 86 Sweden

**Keywords:** SPECT, Quantitative evaluation, Lung diseases, Computer-assisted image analysis, Respiratory function tests

## Abstract

**Background:**

Heterogeneous ventilation in lungs of individuals with allergies, cigarette smokers, asthmatics and chronic obstructive pulmonary disease (COPD) patients has been demonstrated using imaging modalities such as positron emission tomography (PET), magnetic resonance imaging (MRI) and single-photon emission computed tomography (SPECT). These individuals suffer from narrow and/or closed airways to various extents. By calculating regional heterogeneity in lung ventilation SPECT images as the coefficient of variation (CV) in small elements of the lung, heterogeneity maps and CV-density curves can be generated and used to quantitatively measure heterogeneity. This work explores the potential to use such measurements to detect mild ventilation heterogeneities in lung-healthy subjects.

**Method:**

Fourteen healthy subjects without documented lung disease or respiratory symptoms, and two patients with documented airway disease, inhaled on average approximately 90 MBq ^99m^Tc-Technegas immediately prior to the 20-min SPECT acquisition. Variation in activity uptake between subjects was compensated for in resulting CV values. The area under the compensated CV density curve (AUC), for CV values greater than a threshold value CV_T_, AUC(CV > CV_T_), was used as the measure of ventilation heterogeneity.

**Results:**

Patients with lung function abnormalities, according to lung function tests, generated higher AUC(CV > 20%) values compared to healthy subjects (*p* = 0.006). Strong linear correlations with the AUC(CV > 20%) values were found for age (*p* = 0.006) and height (*p* = 0.001). These demonstrated that ventilation heterogeneities increased with age and that they depend on lung size. Strong linear correlations were found for the lung function value related to indices of airway closure/air trapping, residual volume/total lung capacity (RV/TLC; *p* = 0.009), and diffusion capacity of the lung for carbon monoxide adjusted for haemoglobin concentration in the blood (DLCOc; *p* = 0.009), a value partly related to supposed ventilation/perfusion mismatch. These findings support the association between conventional lung function tests and the AUC(CV > 20%) value.

**Conclusions:**

Among the healthy subjects, there is a group with increased AUC(CV > 20%) values, but with normal lung function tests, which implies that it might be possible to differentiate ventilation heterogeneities earlier in a disease process than by lung function tests.

**Electronic supplementary material:**

The online version of this article (doi:10.1186/s13550-014-0039-1) contains supplementary material, which is available to authorized users.

## Background

Single-photon emission computed tomography (SPECT) is increasingly used as a tool in respiratory research and clinical application. Quantitative lung SPECT has been shown to be useful for assessment of regional severity of emphysema [1],[2], aerosol deposition and clearance [3], ventilation and perfusion ratios [2],[4], early radiation-induced lung injury [5], regional perfusion and ventilation [6],[7], and of ventilation heterogeneity [8].

Heterogeneous, uneven or patchy ventilation in the lungs of individuals with allergies, cigarette smokers, asthmatics and COPD patients has been demonstrated using various imaging techniques such as positron emission tomography (PET), magnetic resonance imaging (MRI) and SPECT [9]–[15]. These patients suffer from narrow and/or closed airways by varying degrees. Narrowing of the airways is caused by inflammation, secretions and the shortening of muscle fibres around the bronchial walls, which obstructs airflow. Emami et al. [13] showed that asymptomatic smokers had a more heterogeneous ventilation distribution compared to healthy non-smokers. Patchiness can be caused by narrowing of both larger and smaller airways. Sovijarvi et al. [14] suggested that although larger asthmatic airways are dilated by isoprenaline inhalations, residual bronchial obstruction may still remain in some smaller airways, maintaining a heterogeneous distribution. Furthermore, Tgavalekos et al. [9] concluded that the heterogeneous and patchy distribution of ventilation in asthma patients is a manifestation of the complex behaviour of the airway system, rather than the independent behaviour of individual airways. Closure of the airways by air trapping occurs when the small airways, the bronchioles, collapse. According to Ozer et al. [16], air trapping can be present even before pulmonary function tests reveal abnormal results, or pulmonary symptoms become apparent. Possible causes of air trapping are aging, smoking and various obstructive diseases such as asthma [11],[16]. The severity of air trapping has been shown to increase with age and smoking [17].

Regional heterogeneity in lung ventilation SPECT images may be determined by calculating the coefficient of variation (CV) in small elements of the lung [8],[18]. Heterogeneity maps and density curves can then be generated based on the CV values. This method was used in earlier work, whereby density curves were evaluated using our proposed CV_T_ method [8]. Using the revised method, the proportion of CV values greater than a threshold value CV_T_ may be determined. The purpose of the CV_T_-method is to discriminate between activity distributions in the lungs of healthy subjects and subjects with affected ventilation. The method has been shown to be capable of identifying simulated mild COPD in an anthropomorphic phantom, and to differentiate patients with severe COPD from healthy subjects. The next step is to evaluate the ability of the CV_T_ method to detect genuine abnormalities in subjects who are ‘borderline normal’, according to lung function tests. Since the outcome of a lung function test is a summation of the status of the whole lung, quite significant changes in lung function may occur before a deviation from the norm can be identified. A method that can reliably detect lung abnormalities earlier than lung function tests would therefore be beneficial.

This work explores the potential advantages of quantitative heterogeneity measurements by performing a pilot study of the CV measurements with healthy human subjects, i.e. those without documented lung disease, respiratory symptoms or lung function abnormalities. The outcome is discussed in relation to gender, age, lung size, subtle findings on lung function tests and reported allergies, and smoking history.

## Methods

### Human subjects

Fourteen human subjects were included who were without documented lung disease or respiratory symptoms, and who were more than 40 years old. The 14 subjects were examined by lung SPECT and lung function tests at the Department of Clinical Physiology, Linkoping University Hospital during 2012 to 2013. The subjects were also asked to fill in a health questionnaire. In addition, two patients with documented airway disease or pulmonary symptoms underwent the same examination procedure (referred to as P1 and P2 in Table 1). P1 suffered from asthma and homozygote alfa-1-antitrypsin deficiency and P2 had advanced COPD with emphysema, according to both lung function tests and high-resolution computed tomography (HRCT). Gender, age, height, weight, body mass index (BMI), smoking status, history of allergy and the result of lung function tests for these 16 subjects are presented in Tables 1 and 2. All subjects were informed and written consent was obtained. The regional Ethics Review Board in Linkoping approved the study protocol.

**Table 1:** **Characteristics of the subjects in order of increasing AUC(CV > 20%) value**

Subject	Gender	Age (year)	Height (cm)	Weight (kg)	Body mass index	Smoking status	Passive smoker	Allergy	Airways affected by allergic reaction	Lung function abnormalities (lung function tests)	AUC(CV > 9.5%) [%]	AUC(CV > 20%) [%]
S1	M	49	191	82	22.5	Never	No	Yes^d^	Upper^j^	No^m^	76	29
S2	M	49	185	77	22.5	Never	No	Yes^e^	Upper^j^	No	77	30
S3	M	50	190	85	23.5	Never	No	Yes^f^	Upper^j^	No	78	30
S4	M	48	187	75	21.4	Never	Yes^a^	No	-	No	77	31
S5	M	50	178	72	22.7	Never	No	No	-	No	78	33
S6	M	69	181	100	30.5	Never	Yes^a^	No	-	No	78	34
S7	M	60	188	85	24.0	Current	-	No	-	No^n^	78	34
S8	F	65	177	66	21.1	Current	-	Yes^g^	Lower^k^	No	80	34
S9	F	51	170	85	29.4	Never	Yes^a^	No	-	No	78	36
S10	M	75	175	92	30.0	Never	No	Yes^h^	Upper^j^	No^o^	79	39
S11	M	73	175	77	24.9	Ex-smoker	-	No	-	No	80	41
S12	F	67	172	78	26.4	Never	Yes^b^	No	-	No^p^	83	47
S13	F	67	167	79	28.3	Never	Yes^c^	Yes^i^	Lower^l^	No^q^	84	47
P1	M	55	176	85	27.4	Never	-	-	-	Yes^o,r,s^	83	43
P2	M	81	167	58	20.8	Ex-smoker	-	-	-	Yes	90	61

^a^20 years during childhood; ^b^14 years during childhood and occupational; ^c^10 years occupational; ^d^grass, pollen and dust; ^e^fur animals and grass; ^f^fur animals and pollen; ^g^strong fragrances; ^h^cat and chocolate; ^i^birch pollen, timothy, grass, nuts, penicillin and sulfonamide; ^j^rhinoconjunctivitis; ^k^cough; ^l^shortness of breath; ^m^subject medicated with nasal spray of Budesonide at time of examination; ^n^based on FEV1, FVC, FEV1/FVC, TLC and RV without bronchodilation; ^o^based on DLCOc before and FEV1, FVC, FEV1/FVC, TLC and RV after bronchodilation; ^p^possible early signs of emphysema based on a reduced DLCOc value (see Table 2) and an elevated haemoglobin concentration in the blood; ^q^based on FEV1, FVC and FEV1/FVC without bronchodilation; ^r^subject medicated with inhalation of Budesonide and Formoterol in combination at time of examination; ^s^moderate obstruction and signs of reversibility.

**Table 2:** **Lung function values of subjects S10 to S13 and P1 to P2**

Subject	FEV1 (% pred)	FVC (% pred)	FEV1/FVC (% pred)	TLC (% pred)	RV (%pred)	RV/TLC (%pred)	DLCOc (%pred)	Significant (≥12%) change of FEV1 after bronchodilator	AUC(CV > 20%) [%]
S10	125	119	107	-	-	-	85	No	39
S11	114	114	102	103	96	93	95	No	41
S12	89	91	98	106	128	120	76	No	47
S13	91	94	98	-	-	-	-	-	47
P1	79	107	74	-	-	-	85	Yes	43
P2	40	57	72	132	214	165	38	No	61

Subjects with AUC(CV > 20%) values of more than 37%. Subjects S10 to S13 are listed in order of increasing AUC(CV > 20%) value. FEV1, forced expiratory volume over one second; FVC, forced vital capacity; TLC, total lung capacity; RV, residual volume; DLCOc, single breath dilution carbon monoxide test adjusted for haemoglobin concentration in the blood. Lung function values without bronchodilator use are reported as percentage of predicted reference values (% pred).

### Health questionnaire

The questionnaire covered pulmonary symptoms, smoking history, history of health checks and hospitalisations, allergies, hypersensitivity and medications.

### Lung function tests

The lung function tests consisted of:

Dynamic flow rate and static lung volume measurements before and after inhalation of bronchodilator (Salbutamol, 1.6 alt 0.8 mg):

○ FEV1: forced expiratory volume over 1 s

○ FVC: forced vital capacity

○ RV: residual volume

○ TLC: total lung capacity

Based on these results, the ratios FEV1/FVC and RV/TLC were determined.

DLCOc: gas exchange across alveolar capillary membrane (without bronchodilator). This is measured by means of single breath test of diffusion capacity for carbon monoxide adjusted for haemoglobin concentration in the blood.

The measurements were performed according to the ATS criteria [19]–[21]. The measurements were performed and documented using a Jaeger MasterScreen (Body and Diffusion) (CareFusion Germany 234 GmbH, Hoechberg, Germany). A nose clip was used during all lung function tests. Previously published reference values for gender, age and height were used [22],[23], according to clinical routine. In Table 2, lung function values recorded without previous bronchodilation are given as percentage of predicted reference values, e.g. FEV1 % predicted. One experienced physician evaluated the data of the lung function tests, blinded to the results of this quantitative SPECT-study and also blinded to the identity of the subjects and patients.

### Ventilation SPECT, acquisition and reconstruction

The lung SPECT examinations were performed as described previously [8]. Briefly, starting from functional residual capacity, the subjects took a deep inhale of Technegas and held their breath for 2 to 5 s and then exhaled. If necessary, this procedure was repeated. On average, approximately 90 MBq of Technegas was deposited in the lungs of each subject. The subjects inhaled the gas in a supine position, and a ventilation SPECT was immediately acquired in the same position, using a low-energy high-resolution collimator on a GE Infinia (Milwaukee, WI, USA). A low-dose computed tomography (CT) scan was also performed in the same position using the X-ray source and detector mounted on the gamma camera. The effective dose for this protocol is estimated to be 3.1 mSv (1.3 mSv for SPECT [24] and 2 mSv for CT), which required a total acquisition time of 25 min.

Three-dimensional (3D) SPECT images were reconstructed using the iterative ordered subset expectation maximisation reconstruction software developed at Johns Hopkins University, Baltimore, MD, USA. The reconstruction included correction for attenuation, scatter and collimator detector response (CDR). The CT scans were used for attenuation correction. Scatter correction was performed using the effective source scatter estimation (ESSE) [25],[26]. An analytic, geometrical model for CDR compensation was used. Reconstructions were performed using four iterations and 16 subsets [27]. The side length of the cubic voxels in the reconstructed image was 3.45 mm. The reconstructed images were post-filtered using a Butterworth filter [28] with a cut-off frequency of 0.6 cm^−1^ and a power of 6.

### Defining the extension of the lung and sub-volumes

To define the extension of the lung, a semi-automated procedure was used for each subject based on the individual CT acquisition and an empirical linear attenuation coefficient threshold value of 0.12 cm^−1^. This segmented lung volume was used for analysis unless otherwise stated. An inner volume of interest was created by eroding the outer boundary of the segmented lung by three voxels [29]. The outer volume of interest was created by subtracting the inner volume from the original, segmented lung.

### Method for analysis of heterogeneities

#### The density curve and the CV-based measure

The density curve method applied has been described in detail previously [8]. Briefly, the coefficient of variation (CV) is calculated for overlapping cubic volumes (1 cm^3^) covering the 3D reconstructed activity distribution in the segmented lung, creating a 3D CV matrix. The variation of activity uptake between subjects was compensated for in the calculation of CV values, based on the assumption that each subject had a healthy lung volume and that the healthy volumes in all subjects should generate equivalent CV values (for a detailed description, see the Appendix). The compensated CV values of the matrix were plotted as density curves (normalised CV distributions) with an area under the curve (AUC) of 100%, for each subject. A CV-threshold value was defined, CV_T_. The area under the density curve (AUC) for CV values greater than the threshold value was then calculated for all subjects, i.e. AUC(CV > CV_T_).

### Statistics

Linear regression analysis was used to determine associations between AUC(CV > CV_T_) and the explanatory variables age, height, BMI, smoking status and allergy, respectively. The assumptions for linear regression analysis were verified (normality and constant variance among the residuals). Linear correlations were also determined in the same way for AUC(CV > CV_T_) and each of the lung function values recorded in healthy subjects: RV, RV/TLC and DLCOc. Lung function values recorded without previous bronchodilation and expressed in non-normalised values, i.e. not % predicted, were used to compare with AUC(CV_T_) data, since lung SPECT examinations were performed and expressed accordingly. Furthermore, the relationship between lung function abnormalities (yes or no, according to interpreted lung function tests) and the AUC(CV > CV_T_) value was assessed in the same manner; but in this case, both healthy subjects and patients were included.

## Results

The 13 subjects (S1 to S13) included in the analysis are listed in Table 1, together with corresponding values for patient P1 and P2. One subject, who did not successfully inhale an adequate amount of Technegas, was excluded. In general, the youngest and tallest non-smokers (who never smoked) in the study generated the lowest AUC(CV > CV_T_) values (S1 to S4). Thereafter, the AUC(CV > CV_T_) value increased with age and decreasing height. Finally, the highest AUC(CV > CV_T_) values were found in the subject with signs of lung function abnormalities (subject S12) and in the two patients (P1 and P2) who had documented lung diseases.

**Figure 1:**
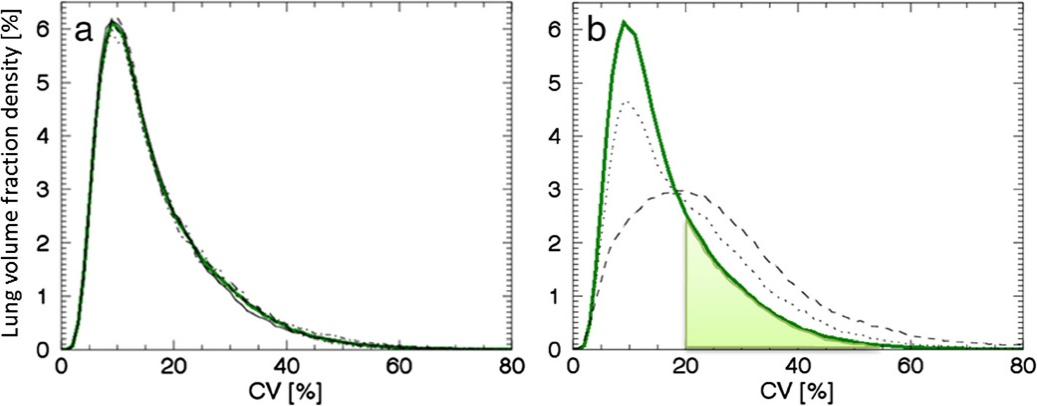
**Density curves for low AUC(CV > 20%) subjects and patients (P1 and P2). (a)** Density curves corresponding to subject S1 (solid line), S2 (dotted line), S3 (dashed line) and S4 (dashed-dotted line), together with their constructed mean curve in green. **(b)** Density curves of patient P1 (dotted line) and P2 (dashed line) together with the mean curve (green line). AUC for CV values greater than 20%, i.e. AUC(CV > 20%), for the mean curve is marked as light green.

The density curves for subjects S1 to S4 were plotted in Figure 1a. A mean density curve was subsequently constructed based on these subjects (hereafter called the mean curve). Subjects S1 to S4 were well represented by the mean curve. These subjects were all males in the same age (48 to 50 years), height (185 to 191 cm) and weight (75 to 85 kg) brackets. All had never smoked, and had normal lung function. Subjects S1 to S3 reported allergies to dust, grass, birch pollen or fur animals, but with manifestations in the upper respiratory tract only (i.e. rhinitis with or without conjunctivitis). Subject S4 reported no allergies. Subject S1 had symptoms of rhinitis, and was medicated with nasal spray of Budesonide, at the time of examination. Based on the mean curve and the CV_T_ method from earlier studies [8],[27], we defined CV_T_ = 9.5% as the mode of the mean curve, and calculated and listed the AUC(CV > 9.5%) for all subjects and patients in Table 1.

The density curves of the patients P1 and P2 are substantially different from the mean curve, as shown in Figure 1b, especially for patient P2, with advanced COPD. The density curve of P1, a patient with documented airway disease, is associated with a larger proportion of high CV values compared to the mean curve, but is not as pronounced as the density curve of P2. Based on Figure 1b, an additional threshold value was defined, CV_T_ *=* 20%, approximately at the point where the patient curves and the mean curve intersect. AUC(CV > 20%) values were calculated for all subjects and patients, and are listed in Table 1. AUC(CV > 20%) for the mean curve is marked as light green in Figure 1b. Since AUC(CV > 20%) resulted in a favourably larger range (29% to 47%) compared to the range of AUC(CV > 9.5%) values (76% to 84%) for the included healthy subjects, CV_T_ = 20% was used hereafter. Listing the subjects in order of increasing AUC(CV > 9.5%) value would introduce shifts between adjacent subjects in Table 1, which implies the chosen threshold has only a minor affect on outcome.

**Figure 2:**
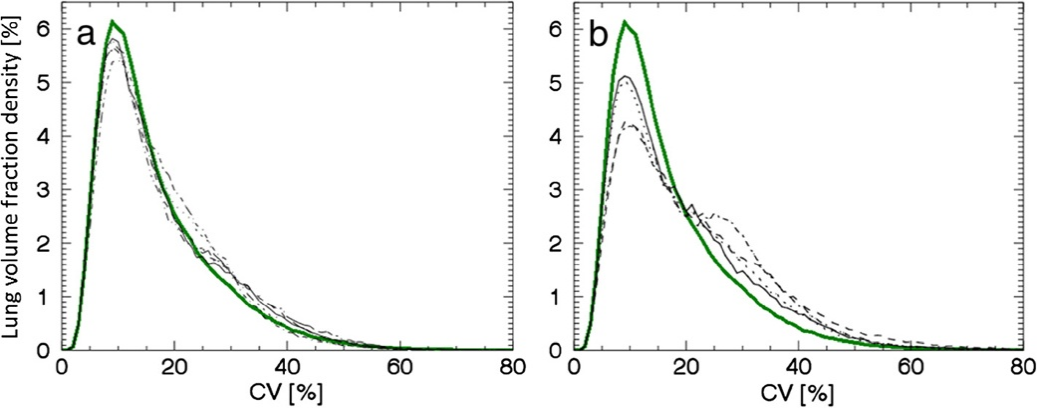
**Density curves for intermediate-AUC(CV > 20%) subjects (S5-S9) and high-AUC(CV > 20%) subjects (S10 to S13). (a)** Five density curves corresponding to intermediate AUC(CV > 20%) values; subject S5 (solid line), S6 (dotted line), S7 (dashed line), S8 (dashed-dotted line) and S9 (dashed-dotted-dotted line), together with the mean curve (green line). **(b)** Four density curves corresponding to the highest AUC(CV > 20%) values of subjects; subject S10 (solid line), S11 (dotted line), S12 (dashed-dotted line) and S13 (dashed line), together with the mean curve (green line).

The remaining healthy subjects were divided into two groups, S5 to S9 and S10 to 13, depending on their density curves, which are shown in Figure 2a,b, respectively. The density curves of subjects S5 to S9 exhibited small differences from the mean curve. This group consisted of subjects with a broader range of characteristics compared to subjects S1 to S4; males and females, aged 50 to 69 years, with a height range of 170 to 188 cm and a weight range of 66 to 100 kg. All subjects in this group had normal lung function, but were current smokers or never smoked and were with or without an allergy. Subject S8 reported hypersensitivity to nickel and strong fragrances. The latter associated with symptoms from the lower respiratory tract (coughing), but at the time of examination, no such symptoms occurred. In contrast, the density curves of subjects S10 to S13 displayed larger differences from the mean curve. This group was also more heterogeneous regarding their characteristics and consisted of males and females, aged 67 to 75 years, with a height range of 167 to 175 cm and a weight range of 77 to 92 kg. They were ex-smokers or had never smoked and were with or without stated allergies. None of these subjects with reported allergies had symptoms at the time of examination. The results of thorough lung function tests were interpreted as normal, with or without early signs of lung function abnormalities. Results of lung function testing of subjects S10 to S13 and P1 and P2 are presented in Table 2.

**Figure 3:**
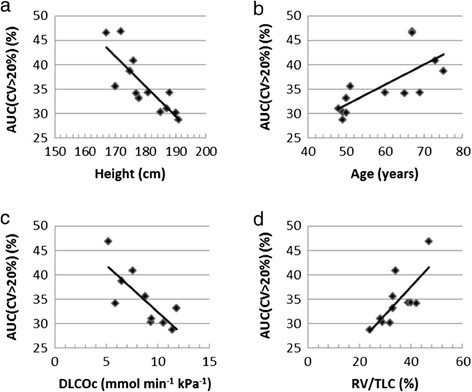
**Correlation between AUC(CV > 20%) and (a) height, (b) age, (c) DLCOc and (d) RV/TLC.** For subjects (S1 to S13). Spread of data to the regression line is shown for each parameter in **(a-d)**.

Significantly higher AUC(CV > 20%) values were found for subjects with abnormal lung function tests compared to those with normal test results (*p* = 0.006). Strong linear correlations were found between the AUC(CV > 20%) value and height and age, respectively, for the 13 healthy subjects, listed in Table 3 and plotted in Figure 3. Sixty-seven percent (see *r*^2^ in Table 3) of the variation in AUC(CV > 20%) can be explained by height and 51% by age. The correlations were statistically significant between AUC(CV > 20%) and both height (*p* = 0.001) and age (*p* = 0.006). For each centimetre increase in height, AUC(CV > 20%) decreased by an average of 0.61 (0.33 to 0.90) units (see B in Table 3). For each additional year of life, the AUC(CV > 20%) value increased by an average of 0.41 (0.15 to 0.68) units. The lung function values RV/TLC and DLCOc were also found to have strong linear correlations with AUC(CV > 20%), also with high statistical significance (*p* = 0.009) (see Table 3 and Figure 3c,d). Ten and 11 of the healthy subjects (in Table 1) had DLCOc and RV/TLC, respectively, measured before treatment with bronchodilator, and were therefore included in the analysis. Males on average had lower AUC(CV > 20%) values than females (*p* = 0.034). No statistically significant differences were found between subjects who never smoked, current smokers and ex-smokers. However, there was a tendency for ex-smokers to have higher AUC(CV > 20%) values compared to non-smokers and current smokers. No difference between healthy subjects with or without stated allergies was observed, but in a sub-group analysis among subjects with allergies, a tendency of higher AUC(CV > 20%) values was found for subjects with symptoms from the lower respiratory tract, compared to the upper respiratory tract. No correlation with AUC(CV > 20%) was found for BMI or RV.

**Table 3:** **Univariate linear regression analysis**

Variables	***r***	B with 95% CI	***r*** ^2^	***p***	***n***
Height (cm)	−0.82	−61 (−90,−33)	0.67	0.001	13
DLCOc (mmol min^−1^ kPa^−1^)	−0.77	−1.9 (−3.2, −0.6)	0.60	0.009	10
RV/TLC (%)	0.74	0.57 (0.18, 0.97)	0.55	0.009	11
Age (years)	0.72	0.41 (0.15,0.68)	0.51	0.006	13

Analysis of the correlation of the AUC(CV > 20%) value with height, age and lung volumes related to closed airways and air trapping (RV/TLC) and ventilation/perfusion mismatch (DLCOc), respectively. Only lung function values obtained without bronchodilator of healthy subjects were used in the analysis. The results are listed in order of decreasing absolute value of the Pearson's correlation coefficient, *r*. B is the gradient of the regression line, given together with lower and upper boundary of the 95% confidence interval. The coefficient of determination, *r*^2^, the associated significance value, *p*, and the number of healthy subjects, *n*, included in the calculations are also included.

**Figure 4:**
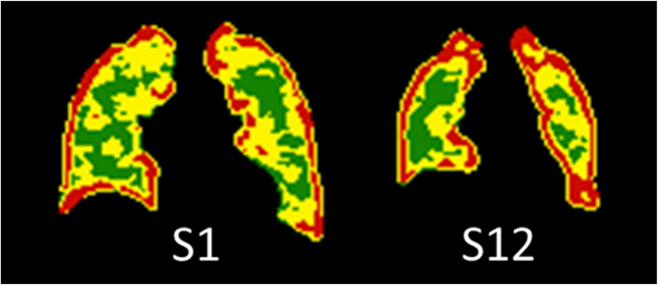
**Coronal slices of CV matrices for subjects S1 and S12.** CV slices with CV values below 9.5% in green, between 9.5% and 20% in yellow and above 20% in red for subject S1 to the left and subject S12 to the right.

One of the strengths of image-based methods is the ability to visualise where quantified heterogeneities are situated. To illustrate where different CV values are generally positioned, we show CV values below 9.5% as green, between 9.5% and 20% as yellow and above 20% in red, for two subjects (Figure 4). Areas in green and yellow are in the peak of the mean curve and are found in the centre of the lung for both subjects. Due to the moderate spatial resolution of the gamma camera system, (false) high CV values are generated in the periphery of the lung of a healthy subject (the periphery or edge effect [8]). Therefore, red areas are found in the periphery of the lungs of both subjects, but a larger proportion is found in subject S12 compared to subject S1.

**Figure 5:**
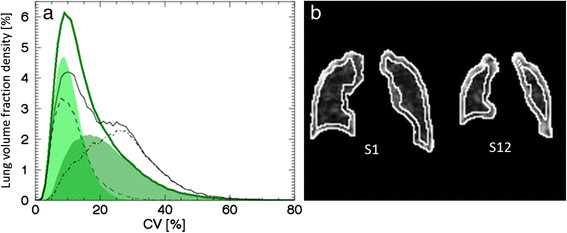
**Entire, inner, peripheral density curves for mean (S1 to S4) and S12, and coronal inner/peripheral contours.** The segmented lung divided into inner and outer volumes, **(a)** the mean density curve (solid green line), and its inner (light green area), and outer (dark green area) volume components, as well as the S12 density curve (solid black line), and its inner (black dashed line) and outer (black dash-dotted line) volume components. **(b)** Coronal slices of the CV matrix for S1 (left, used to calculate the mean) and S12 (right). Contours indicate the inner and outer volumes.

To illustrate the differences in the CV distributions of the inner and outer volume of the segmented lung for subject S12, we created two density curves based on each sub-volume. For comparison, the same procedure was applied to the mean density curve (Figure 5). The main difference in the CV distributions of the mean curve for subjects S1 to S4 and S12 is a shift of CV values in the outer volume, but not in the inner volume. For subject S12, the outer volume is 60% of the total volume, and 50%, 51%, 53% and 56% for the subjects used to calculate the mean curve.

## Discussion

In this study, we used a previously reported CV_T_ method [8] and introduced an adaptation that compensates for subject-to-subject variation, making the CV values from healthy lung volumes of different subjects comparable. This adaptation makes it possible to better evaluate the ability of the CV_T_ method to discriminate between subjects with less pronounced differences in ventilation heterogeneity. In a previous study, we showed that significantly higher AUC(>mode) values resulted from patients with severe COPD, compared to healthy subjects who had never smoked [8]. Therefore, we expect subjects with gradually less altered lung function to generate gradually lower AUC(CV > 20%) values. We found the result of this pilot study very promising since the list of subjects placed in order of increasing AUC(CV > 20%) value (Table 1), calculated with the improved method, supports this idea.

In this study, we have shown that patients (P1 and P2) generated significant higher AUC(CV > 20%) values compared to healthy subjects (S1 to S13) (*p* = 0.006), which is consistent with the result of a previous study [8]. Subjects S10 to S13 showed deviating density curves compared to the mean curve (calculated from subjects S1 to S4), and consequently higher AUC(CV > 20%) values, without abnormalities detected by lung function tests. This implies that it might be possible to detect ventilation heterogeneities by the AUC(CV_T_) method earlier in a disease process than by lung function tests. The fact that one of the 13 healthy subjects, who exhibited possible early signs of emphysema (S12), also showed abnormal AUC(CV > 20%), tends to support the notion that the AUC(CV_T_) method is more sensitive to minor pulmonary abnormalities than conventional lung function tests.

Despite the low sample size in this pilot study, a group of subjects was identified who never smoked and were of the same gender (male), age bracket (48 to 50 years) and physique (height 185 to 191 cm and weight 75 to 85 kg), namely subjects S1 to S4 (Table 1). These subjects displayed the lowest AUC(CV > 20%) values. Three of them (S1 to S3) had allergies with symptoms from the nose only, i.e. the upper respiratory tract, occurring seasonally or after allergen exposure. Therefore, we assume that the reported allergies had no impact on the outcome of the lung function tests or lung SPECT examinations. Subjects S2 and S3 had no symptoms at the time of examination; however, S1 suffered from persistent but subtle symptoms (rhinitis) and this was treated with nasal spray of a corticosteroid at the time of spirometry and lung SPECT examinations.

Lung function values are normalised to a reference distribution depending on gender, age and height. This study has shown that AUC(CV > 20%) also has a dependence on age and height.

Aging is associated with increased chest wall stiffness [30] and an increase in residual volume, which reduces the vital capacity. The effect of aging on lung tissue is an increase in lung compliance, which results in reduced parenchymal recoil on the airways, reducing airway calibre and increasing airway closure and gas trapping [16],[17],[30]. Such changes would result in a more uneven ventilation distribution in an older, healthy subject, compared to a younger, healthy one, which corresponds to an increasing AUC(CV > 20%) value with age. The proposed method confirms this expected increase in AUC(CV > 20%) with age, compatible with the notion of age-dependent alterations of lung compliance (see Table 3).

The geometrical resolution of the SPECT system is limited; and therefore, high CV values will always be found in the periphery of the lung, even in subjects with a healthy lung (Figure 4). This periphery effect results in a size dependence of the AUC(CV > 20%) value, since the peripheral volume of the small lungs is a larger proportion of the total lung volume, compared to the larger lungs (cf. Figure 4). Therefore, small lungs would generate a larger proportion of high AUC(CV > 20%) values compared to larger lungs. Generally, a short person has smaller lungs than that of a taller person. This study demonstrated the expected decrease of AUC(CV > 20%) values with increasing height (see Table 3). Since the lung SPECT examination is performed with the subject in supine position, the volume of the lung should also depend on how much pressure is placed on the diaphragm by the abdomen. This effect would be greater for subjects with a larger BMI. This study, however, found no BMI dependence.

Generally, as in this study, males are taller than females, which might explain why, on average, males resulted in lower AUC(CV > 20%) values than females. The possibility to analyse the outer part of the lungs separately makes it possible to discriminate between healthy and non-healthy distributions in this region (see Figures 4 and 5).

We found a strong correlation between AUC(CV > 20%) and RV/TLC, as well as between AUC(CV > 20%) and DLCOc (see Table 3). The former supports the value of measuring heterogeneities in ventilation lung SPECT images, since this lung function parameter measure the volume with closed airways and air trapping (RV/TLC). The fact that all CV-density curves are normalised to generate the same total area under the curve (100%), might explain why RV/TLC, but not RV per se, showed a strong association with AUC(CV > 20%). One might argue that these correlation studies would speak against the value of lung-SPECT measurements in comparison to lung function testing. However, in this context, it is important to note that all except one of the subjects (S12) was considered fully ‘normal’ by a physician, who is a recognised expert on lung function tests. Furthermore, lung SPECT provides the possibility to visualise the location of the malfunctioning volumes in the lungs (see Figures 4 and 5), unlike lung function tests.

Smoking may lead to increased sputum production, decreased ciliary movement, inflammation of both large and small airways, constriction of the encircling smooth muscle and collapsed airways, resulting in obstructed airways and air trapping [16],[17]. Also, involuntary exposure of non-smokers to tobacco smoke has a negative effect on the lungs, especially for exposed children [31]. Such changes would result in a more uneven ventilation distribution compared to healthy subjects who never smoked [13] and therefore an increased AUC(CV > 20%) value. In this small pilot study, we found a tendency of ex-smokers to have a higher AUC(CV > 20%) value than subjects who never smoked and current smokers.

Allergies with symptoms in the upper respiratory tract, such as allergic rhinitis, is not expected to affect the AUC(CV > 20%) value, while allergies with symptoms in the lower respiratory tract may well, at least during allergy season or after allergen exposure. According to Hens and Hellings, the increasing recognition over recent decades that allergic rhinitis and allergic asthma frequently co-exist has led to the concept of ‘united airways’, and that these disorders are manifestations of the same disease expressed to a greater or lesser extent in either the upper or the lower airways [32]. In some patients, rhinitis predominates and asthma is undiagnosed or sub-clinical; in others, the situation is reversed, while in many cases, both are clinically expressed [33]. In a sub-group analysis among subjects with allergies, a tendency of higher AUC(CV > 20%) values was found for subjects with symptoms from the lower respiratory tract compared to that of the upper respiratory tract.

In this work, Technegas was inhaled with a single full inhalation, but subsequent imaging was performed with the subject breathing normally in supine position. This resulted in a higher activity region in the posterior parts of the lung; the so called ‘rind effect’, caused by gravity compressing the posterior airways that increased the measured CV values in this region. Schembri et al. [34] noted that the ‘rind effect’ was reduced when imaging was done in prone position and with tidal breathing during ventilation. Inhaling the Technegas as a normal breath (with a 5-s breath-hold) might therefore reduce this ‘rind effect’ and thus its effect on the CV values. No attempt was made to exclude possible ‘hot spots’ caused by turbulence in the conducting airways, since ‘hot spots’ are rare in healthy subjects.

One limitation of this study is the low number of participants, and consequently a single observation had a large impact on the linear correlation. With a larger number of subjects with different smoking histories and subjects with allergy symptoms from both the upper and lower respiratory tract, the tendencies found in this pilot study might find statistical significance. The information of possible air trapping and emphysema from HRCT could have been beneficial for this study. The relatively high effective dose from this procedure prevented us from performing HRCT on the healthy subjects.

## Conclusion

Using the adapted CV_T_ method, with the newly implemented compensation technique, we showed that patients with lung function abnormalities detected by conventional lung function tests generated significantly higher AUC(CV > 20%) values compared to healthy subjects with normal lung function tests (*p* = 0.006). Increased AUC(CV > 20%) values were also detected among subjects who had normal lung function tests but indications of conditions associated with ventilation disturbances. The results suggest that our present SPECT method has the capacity to identify minor lung function abnormalities earlier in a disease process than conventional lung function tests. Our positive findings have provided the motivation to extend this pilot study to a full study with a larger number of subjects.

## Appendix

### The compensation method

**Figure 6:**
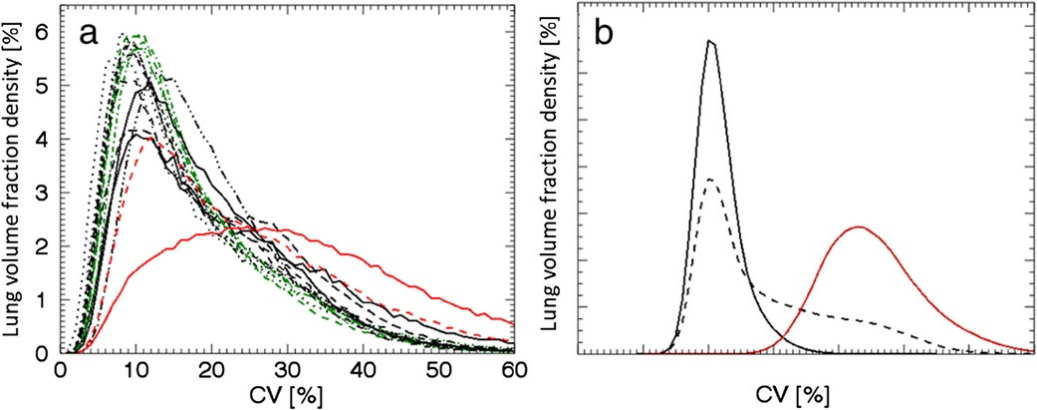
**Density curves, uncompensated for subjects and simulated for phantom activity distributions. (a)** Density curves of the 14 examined healthy subjects: S1 to S4 (green) and S5 to S13 (black), together with the excluded subject (black dash-dott-dott-dotted) and the two patients (red). **(b)** Density curves based on the phantom lung activity distributions that were entirely uniform (solid black line), partly uniform/partly heterogeneous (dotted black line) (with equal activity concentration in the healthy, uniform regions), and entirely heterogeneous (red line).

Our main objective was to adapt an existing method, which is based on the coefficient of variation, CV, to be able to differentiate between varying grades of ventilation heterogeneities in healthy human subjects, according to lung function tests. In both this study and earlier work [8], it was found that for healthy subjects, the positioning of the calculated CV density curves on the CV axis varied to such an extent that it was impossible to discriminate between small heterogeneity variations between subjects (see Figure 6a). Monte Carlo simulations and various predefined activity distributions in a phantom lung were used to analyse properties of the density curve. We tested the density curves for different types of distributions, based on either an entirely uniform (homogeneous) activity distribution, or a partly uniform/partly heterogeneous activity distribution. We noticed that they had the same starting point on the CV axis and the same mode value of their density peaks, provided that the number of ^99m^Tc particles per unit volume in the uniform (‘healthy’) parts of the lungs was the same (see Figure 6b). A simulated density curve of an activity distribution with no uniform regions does not show this pattern (see red line in Figure 6b). The density curves in Figure 6b also illustrate that the low CV values, positioned in the density peaks of the ‘healthy’ and ‘partly healthy’ lungs (black lines in Figure 6b) correspond to the uniform part of the activity distributions, while the heterogeneous parts of the activity distributions are represented by higher CV values.

The CV values for a uniform activity distribution depend on the number of ^99m^Tc particles per unit volume (activity concentration). Density curves for the central voxels (with the periphery effect excluded) of two uniform activity distributions with different concentration levels can be transformed to the same shape and position on the CV axis as that corresponding to the highest activity concentration. This is done by multiplying the CV values of the density curve for the lower activity concentration by the ratio of the mode values of the respective density curves (see Equation 1).

1 $$A_{\mathrm{high}\;T99mc\;\mathrm{concentration}}=A_{\mathrm{low}\;T99mc\;\mathrm{concentration}}\cdot \frac{M_{\mathrm{high}\;T99mc\;\mathrm{concentration}}}{M_{\mathrm{low}\;T99mc\;\mathrm{concentration}}}$$

where *A* is the vector of CV values generating the density curve and *M* is the mode value. The indices ‘high’ and ‘low’ indicate the level of activity concentration.

**Figure 7:**
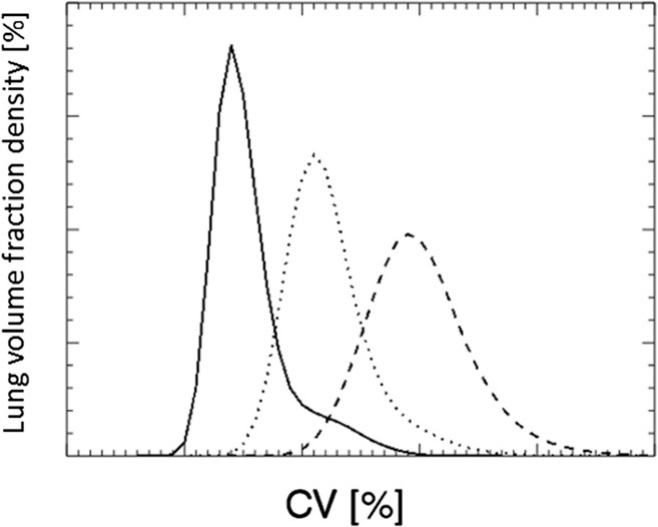
**Density curves for a phantom lung with heterogeneities, with decreasing activity levels.** Density curves based on three decreasing average number of ^99m^Tc particles per unit volume are shown, with the highest (left solid line), middle (centre dotted line) and the lowest (right dashed line).

A phantom lung was used with spherical regions of reduced activity, compared to the surroundings [8]. The original density curves are asymmetric, with a distinct peak at low CV values (corresponding to the healthy parts of the lung) and a tail at higher CV values (corresponding to the heterogeneities). By reducing the activity, the density curve becomes a symmetric bell (Figure 7). This change in shape is caused by the gradual ‘loss of information’ by the SPECT system due to the increase in statistical noise. If Equation 1 were applied to transform the density curve based on a low to a higher average particle concentration, it would not fully succeed. This is because the tail end, containing the heterogeneity information, cannot be restored (see Figure 7); the transformed CV distribution would look like that of a uniform activity distribution. Furthermore, when transforming a density curve based on a high to a lower average particle concentration, the resulting density curve would contain too long a tail of CV values. Therefore, any transformation has to be performed with caution.

The compensation method we propose is an attempt to compensate for subject-to-subject variation in activity uptake, and is based on the observations described above. The basic assumption is that each subject has a healthy lung volume that is large enough to generate a distinct peak in the density curve. Since the aim of the study was to find mild ventilation heterogeneities in subjects with normal lung function, according to lung function tests, we assume that subjects with no healthy lung regions are rare.

The compensation procedure was performed in two steps. First, a shift of all density curves was made so that the left flank of the density curves passed through a well-chosen point in the plot, namely a density of 0.5% at 3 CV% (hereafter called the fix point). This point was chosen because many of the uncompensated density curves passed close to this point (Figure 6a). A hypothetical healthy subject with a density curve resembling the mean curve, as defined in the results of this study, that intersects the fixed point is referred to here as the norm. This curve needs no compensation. Secondly, a transformation of the shape of the density curves was performed according to Equation 2.

2 $$A_{\mathrm{compensated}}\;=\;A_{\mathrm{shifted}}\cdot \frac{9.5}{9.5‐\mathrm{shift}}$$

where *A*_compensated_ is the vector of CV values generating the compensated density curve and *A*_shifted_ is the shifted CV vector. The mode value of the mean curve defined in the ‘Results’ section is 9.5%. The shift is negative if the subject has a lower activity concentration in its healthy regions compared to the norm.

**Figure 8:**
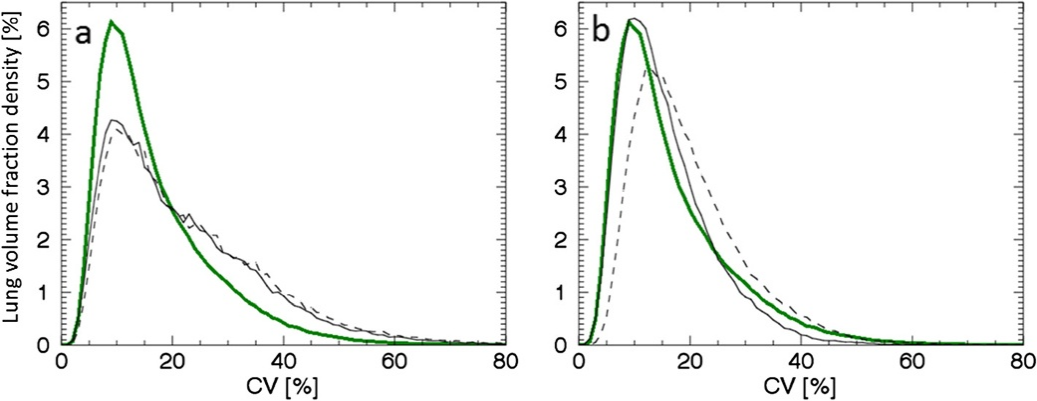
**Density curves before and after compensation for (a) S13 and (b) S (excluded); both with mean (S1 to S4).** For both subjects, curves are shown for before (black dashed line), after (black solid line) compensation, together with the compensated mean curve (green solid line). The subject in **(b)** was excluded due to a relatively low concentration of Tc particles per unit volume in the lung.

Compensations according to Equation 2 result in less pronounced changes in the shape of the density curve compared to using Equation 1. The density curves of subject S13, before and after compensation, are shown in Figure 8a. In this case, the effect of the compensation was small. The effect of the compensation can sometimes be too large, even though we have chosen a compensation method that has a lower impact on the density curve compared to that using Equation 1. Therefore, there is a limiting minimum activity concentration for which the compensation method is useful. This is illustrated in Figure 8b. The subject was excluded from the study due to a relatively low concentration of ^99m^Tc particles in the lungs. When the proposed compensation method was applied to the density function of this subject, the resulting AUC(CV > 20%) value was even lower than that for the subjects representing the mean curve. This is a misleading result.

## Authors’ original submitted files for images

Below are the links to the authors’ original submitted files for images. Authors’ original file for figure 1 Authors’ original file for figure 2 Authors’ original file for figure 3 Authors’ original file for figure 4 Authors’ original file for figure 5 Authors’ original file for figure 6 Authors’ original file for figure 7 Authors’ original file for figure 8

## Abbreviations

AUC: area under the curve
AUC(CV_T_): area under the curve for CV values greater than CV_T_
BMI: body mass index
CDR: collimator-detector response
COPD: chronic obstructive pulmonary disease
CV: coefficient of variation
CV_T_: CV threshold value
DLCOc: diffusion capacity of the lung for carbon monoxide adjusted for haemoglobin concentration in the blood
ESSE: effective source scatter estimation
FEV1: forced expiratory volume in one second
HRCT: high-resolution computed tomography
RV: residual volume
SPECT: single-photon emission computed tomography
TLC: total lung capacity
FVC: forced vital capacity
