# Supplementary figures and images for: Does quantitative lung SPECT detect lung abnormalities earlier than lung function tests? Results of a pilot study
Source: EJNMMI Res. 2014 Aug 1;4:39. doi: 10.1186/s13550-014-0039-1 (PMC4884006; doi:10.1186/s13550-014-0039-1)

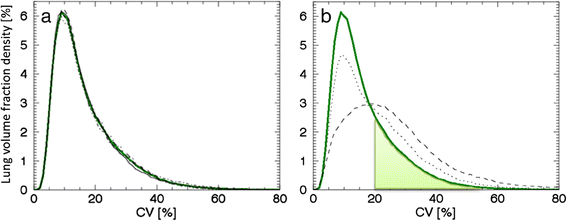

Supplement: Supplementary file 1 — Authors’ original file for figure 1 [file 13550_2014_39_MOESM1_ESM.gif]

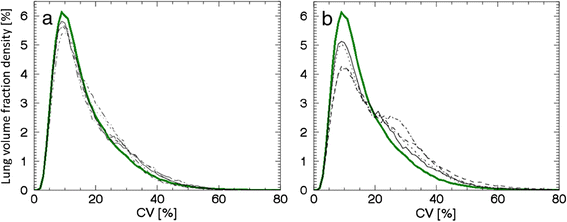

Supplement: Supplementary file 2 — Authors’ original file for figure 2 [file 13550_2014_39_MOESM2_ESM.gif]

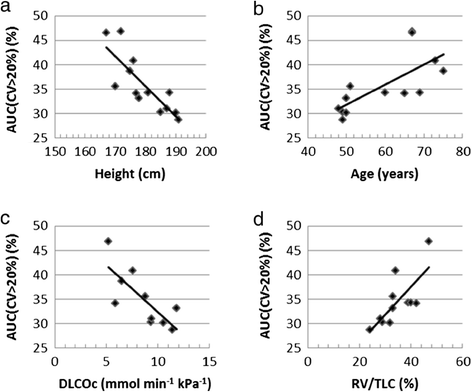

Supplement: Supplementary file 3 — Authors’ original file for figure 3 [file 13550_2014_39_MOESM3_ESM.gif]

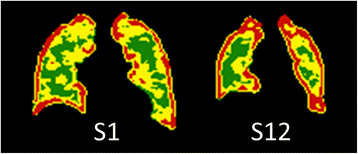

Supplement: Supplementary file 4 — Authors’ original file for figure 4 [file 13550_2014_39_MOESM4_ESM.gif]

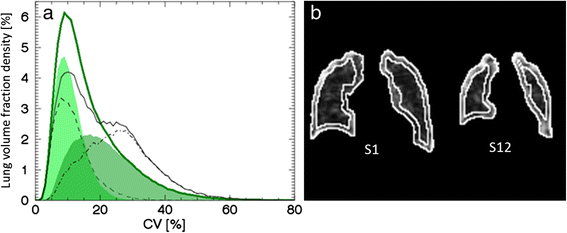

Supplement: Supplementary file 5 — Authors’ original file for figure 5 [file 13550_2014_39_MOESM5_ESM.gif]

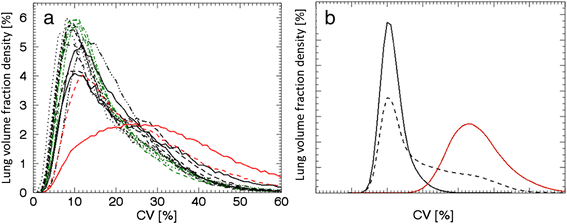

Supplement: Supplementary file 6 — Authors’ original file for figure 6 [file 13550_2014_39_MOESM6_ESM.gif]

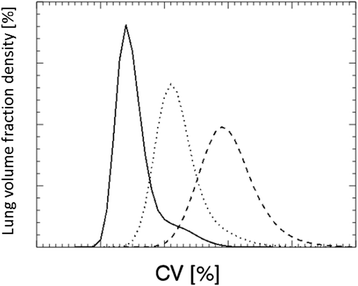

Supplement: Supplementary file 7 — Authors’ original file for figure 7 [file 13550_2014_39_MOESM7_ESM.gif]

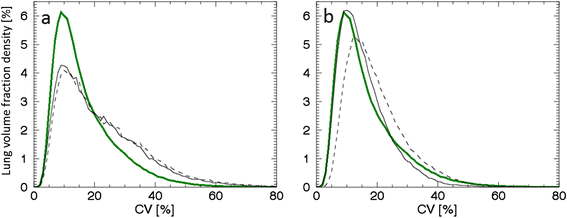

Supplement: Supplementary file 8 — Authors’ original file for figure 8 [file 13550_2014_39_MOESM8_ESM.gif]
